# Characterization and expression analysis of MATEs in *Cannabis sativa* L. reveals genes involving in cannabinoid synthesis

**DOI:** 10.3389/fpls.2022.1021088

**Published:** 2022-10-13

**Authors:** Sifan Wang, Xue Cao, Xiangxiao Meng, Maimaiti Aili, Qin Dou, Yan Wang, Atia Tul Wahab, Shilin Chen, Wei Sun, Huihua Wan, Weiqiang Chen

**Affiliations:** ^1^ Key Laboratory of Beijing for Identification and Safety Evaluation of Chinese Medicine, Institute of Chinese Materia Medica, China Academy of Chinese Medical Sciences, Beijing, China; ^2^ Xinjiang Institute of Traditional Uyghur Medicine, Urumqi, China; ^3^ Hussain Ebrahim Jamal Research Institute of Chemistry, International Center for Chemical and Biological Sciences, University of Karachi, Karachi, Pakistan; ^4^ Panjwani Center for Molecular Medicine and Drug Research, International Center for Chemical and Biological Sciences, University of Karachi, Karachi, Pakistan

**Keywords:** *Cannabis sativa*, cannabinoids, transporter, MATEs, genome-wide, heterologous biosynthesis

## Abstract

The medicinal plant *Cannabis sativa* L. (*C. sativa*) accumulates plant cytotoxic but medicinally important cannabinoids in glandular trichomes and flowers of female plants. Although the major biosynthetic pathway of cannabinoids has been revealed, their transportation mechanism is still unknown. Multidrug and toxic compound extrusion proteins (MATEs) can transport plant metabolites, ions and phytohormones intra and inter-cellularly. MATEs could have the potential to translocate cannabinoids or their synthetic intermediates to cellular compartment, thus protecting them from unwanted modifications and cytotoxicity. In this study, we performed a genome-wide identification and expression analysis of *Cannabis sativa* MATEs (CsMATEs) and revealed 42 CsMATEs that were classified phylogenetically into four conserved subfamilies. Forty-two *CsMATEs* were unevenly distributed on 10 chromosomes, with 50% *CsMATEs* were physically adjacent to at least one another *CsMATEs* and 83% CsMATEs localized on plasma membrane. Tandem duplication is the major evolutionary driving force for *CsMATEs* expansion. Real-time quantitative PCR revealed *CsMATE23*, *CsMATE28* and *CsMATE34* mainly expressed in flower, whereas *CsMATE17* and *CsMATE27* showed strong transcription in root. Light responsive *cis*-acting element was most abundant in promoters of *CsMATE23*, *CsMATE28* and *CsMATE34*. Finally, the contents of cannabinoids and corresponding biosynthetic intermediates as well as expressions of *CsMATE28* and *CsMATE34* were determined under UV-B treatment, among which strong correlation was found. Our results indicates that CsMATEs might involve in biosynthesis of cannabinoids and has the potential to be used in heterologous production of cannabinoids.

## Introduction

The annual dioecious herb *Cannabis sativa* L. (*C. sativa*) from *Cannabis* family accumulates terpenophenolic cannabinoids in glandular trichomes and flowers of female plants (Andre et al., 2016). Till now, over 113 cannabinoids have been isolated from *C. sativa* and many are derived from non-enzymatic decarboxylaion of their acidic forms by heat or UV irradiation (Güllck and Moller, 2020). The well-studied cannabinoids cannabigerol (CBG), Δ9-tetrahydrocannabinol (THC), cannabidiol (CBD) and cannabichromene (CBC) share the same biosynthetic precursors of olivetolic acid (OA) and geranyl diphosphate (GPP) (Güllck and Moller, 2020). Phenolic OA is condensed from one molecule of hexanoyl-CoA and three molecules of malonyl-CoA by coordinated work of olivetol synthase (OLS) (Taura et al., 2009) and olivetolic acid synthase (OAC) (Gagne et al., 2012) in cytosol. The acyl-activating enzyme 1 (AAE1) provides hexanoyl-CoA by converting C6-hexanoic acid which is derived from fatty acid pathway (Stout et al., 2012), while C10-isoprenoid GPP is synthesized by 2-C-methyl-D-erythritol 4-phosphate (MEP) pathway in plastid (Fellermeier et al., 2001). Aromatic prenyltransferase 4 (aPT4) from UbiA protein superfamily subsequently prenylates OA by GPP to produce cannabichromenic acid (CBGA) (Luo et al., 2019; Güllck et al., 2020), which is further oxidized to CBG. CBGA could be converted to Δ9-tetrahydrocannabinolic acid (THCA) or cannabidiolic acid (CBDA) by flavoproteins Δ9-tetrahydrocannabinolic acid synthase (THCAS) (Sirikantaramas et al., 2005) or cannabidiolic acid synthase (CBDAS) (Taura et al., 2007), respectively, and subsequently decarboxylated to THC or CBD by light or heat.

Low abundance in *C. sativa* and important pharmaceutical use evokes tremendous interests for heterologous biosynthesis of cannabinoids. A notable example is the *de novo* production of THCA and CBDA in yeast *Saccharomyces cerevisiae* system (Luo et al., 2019). Co-expression of OA synthetic pathway (*CsAAE1*, *CsOAC* and *CsOLS*) along with plastid-localization-signal-free *CsaPT4*, secretory-signal-free *CsTHCAS* or *CsCBDAS* in a GPP-overproducing yeast strain yielded THCA or CBDA, respectively. When those genes were transiently expressed in tobacco (*Nicotiana benthamiana*) cells, however, either OA, CBGA or THCA was severely glucosylated (Güllck et al., 2020). In addition to been easily glucosylated, CBGA and THCA are cytotoxic (Morimoto et al., 2007). Therefore, mechanisms might exist in *C. sativa* cells that protect cannabinoids from glucosylation and inhibit their cytotoxicity.

Multidrug and toxic compound extrusion proteins (MATEs) are known to translocate second metabolites, ions, and phytohormones intra and intercellularly, and are associated with exnobiotic efflux, aluminum detoxification and disease resistance (Upadhyay et al., 2019). Moreover, MATEs are widely distributed in prokaryotes and eukaryotes, including plants. YdhE was the first isolated MATE from *E.coli* as a multidrug efflux protein that confers bacteria drug resistance (Morita et al., 1998). Soon after, ALF, the first plant MATE from *Arabidopsis thaliana* (*A. thaliana*) (Diener et al., 2001) and many other plant MATEs were sequentially discovered (Wang et al., 2016; Santos et al., 2017; Wang et al., 2017; Huang et al., 2021). Most MATEs consist of about 400-700 amino acids with 12 transmembrane helices and a common MatE domain (pfam 01554), but no consensus sequence thus far has been found in all MATEs (Kusakizako et al., 2020). MATEs transport second metabolites into and out of the membranes through the employment of either Na^+^ or H^+^ electrochemical gradient (Hvorup et al., 2003). For instance, NtMATE1 and NtMATE2 that localize on the vacuolar membrane of tobacco root cells are believed to sequestrate alkaloid nicotine within the vacuole of the roots, then nicotine is transported to the above-ground organs of the plant in response to plant biotic stresses (Shoji et al., 2009). Similar function in alkaloid transportation is also found for tobacco MATEs Nt-JAT1 and Nt-JAT2, which localize on the vacuolar membrane of leaf cells (Morita et al., 2009). GhTT2, GhMATE12, GhMATE16 and GhMATE38 from *Gossypium hirsutum* L. localized on the tonoplast are proposed to translocate phenolic pro-anthocyanidins (Gao et al., 2016; Xu et al., 2019). VvAM1 and VvAM2 expressed in berry skins of *Vitis vinifera* and subcellularly localized on tonoplast are responsible of translocating flavonoid anthocyanins (Perez-Diaz et al., 2014). Thus, we hypothesis that MATEs from *C. sativa* might have the ability to bind and translocate terpenophenolic cannabinoids or corresponding biosynthetic intermediates, protecting them from glucosylation and inhibiting their cytotoxicity in *C. sativa* cells.

In this study, we identified 42 MATEs in *C. sativa* genome and characterized their physical-chemical properties, gene structure, motif composition and gene expression patterns. Furthermore, qRT-PCR verification and *cis*-acting elements analysis of *CsMATEs* that predominantly expressed in cannabinoids accumulation tissues were conducted. In addition, effects of UV-B on accumulation of cannabinoids and corresponding biosynthetic substrates as well as the expression of *CsMATE23*, *CsMATE28* and *CsMATE34* were studied. Together, our result indicates that CsMATE28 and CsMATE34 might be involved in biosynthesis of cannabinoids or its biosynthetic intermediates.

## Materials and methods

### Plant material and growing conditions

In this study, we used the *C. sativa* variety Dinamed Kush (DK) for transcriptome sequencing (Yang et al., 2021). DK were grown in the experimental field of the Institute of Chinese Materia Medica of the Chinese Academy of Chinese Medical Sciences, China. Three-weeks-old *C.* s*ativa* grown in green house at 65% humidity and light wavelength of 380-780 nm (520-860 uM m^-2^ s^-1^) was used for UV-B light treatment.

### Date source

Genome of female *C. sativa* CRBRx (GCA_900626175.1) and transcriptional data of other nine *C. sativa* varieties (Zager et al., 2019) were obtained from the NCBI database (https://www.ncbi.nlm.nih.gov). *Arabidopsis thaliana* MATE gene family members were obtained from the Uniprot database (www.uniprot.org).

### Identification and basic information of CsMATEs

CDS sequences and protein sequences of CRBRx were extracted using TBtools (Chen et al., 2020). Protein sequences of *A. thaliana* MATEs were used as queries to perform homology search by BLASTp method (score value of ≥100, e-value ≤ e^−10^). Duplicated proteins were manually removed. Recognizable domains were initially retrieved using BLAST-based NCBI conserved domain searches (https://www.ncbi.nlm.nih.gov/Structure/cdd/wrpsb.cgi). Molecular weights and isoelectric points were predicted using ExPASy (Duvaud et al., 2021). Subcellular localizations were predicted by WOLF PSORT (Horton et al., 2007).

### Chromosomal location, gene structure, phylogenic tree, conserved motifs and *cis*-acting elements analysis of CsMATEs

Visualization of *CsMATEs* structures and chromosome locations were conducted using TBtools (Chen et al., 2020). The amino acid sequences were aligned using MEGA 7.0 software (Kumar et al., 2016). Phylogenetic trees of *C. sativa* and *A. thaliana* were constructed using the neighborjoining (NJ) method with 1000 bootstrap replicates. Conserved motif information of CsMATEs was analyzed using MEME (Bailey et al., 2006). *Cis*-acting elements in 2000 bp upstream of the start codon of each *CsMATEs* were analyzed using PlantCARE (Lescot et al., 2002).

### Transcriptomic data analysis, alternative splicing and qRT-PCR

mRNA from five different tissues or organs of female *C. sativa* Dinamed Kush were sequenced. TBtools (Chen et al., 2020) was used to analyze and visualize the differential expression data of *CsMATEs*. RNA was extracted using a kit (Tiangen Biotech, Beijing, China) according to manufacturer’s instructions. Three biological replicates of each sample were performed. Extracted RNA was examined by agarose gel electrophoresis and concentrations were determined using Nanodrop (Thermo fisher scientific, Beijing, China). cDNA synthesis was performed using the Reverse Transcription Kit (TransGen, Beijing, China) as described in instruction. qRT-PCR was designed using NCBI-Primer blast (**Supplementary Table 1**) with *EF1-α* as the reference gene. qRT-PCR reaction included StarLighter SYBR green qPCR mix (Qi Heng Xing, Beijing, China) 10 μL, cDNA template 1 μL, 0.4 μL of each primer and ddH2O 8.2 μL. The CFX96™ real-time system (Roter-Gene Q MDx, QIAGENBio-Rad, Germany) was used for qRT-PCR. The reaction conditions were: 95°C for 5 min, 35 cycles of 95°C for 30 s, 55°C for 30 s, and 72°C for 90 s. Data were processed using 2^-ΔΔCT^ (Livak and Schmittgen, 2001).

### QQQ-MS/MS conditions

Cannabinoid content was determined using an Agilent UPLC 1290II-G6400 triple quadrupole mass spectrometer (QQQ; Agilent Technologies, Santa Clara, CA). The autosampler was set to 4°C and a 3-μl sample volume was injected. The chromatographic column was a (2.1 * 100 mm, 1.8 μm) C18 column. Mobile phase A contained water with 0.1% formic acid; phase B was 100% methanol. Elution was performed at 0.3 mL/min.

### Statistical analysis

All the data were analyzed using Prism 8 Statistics programs. One-way analysis of variance (ANOVA) followed by Tukey’s multiple range test was used both for metabolic data and gene expression data.

## Results

### Identification, characterization and phylogenic analysis of the *MATE* genes in *C. sativa*

**Figure 1:**
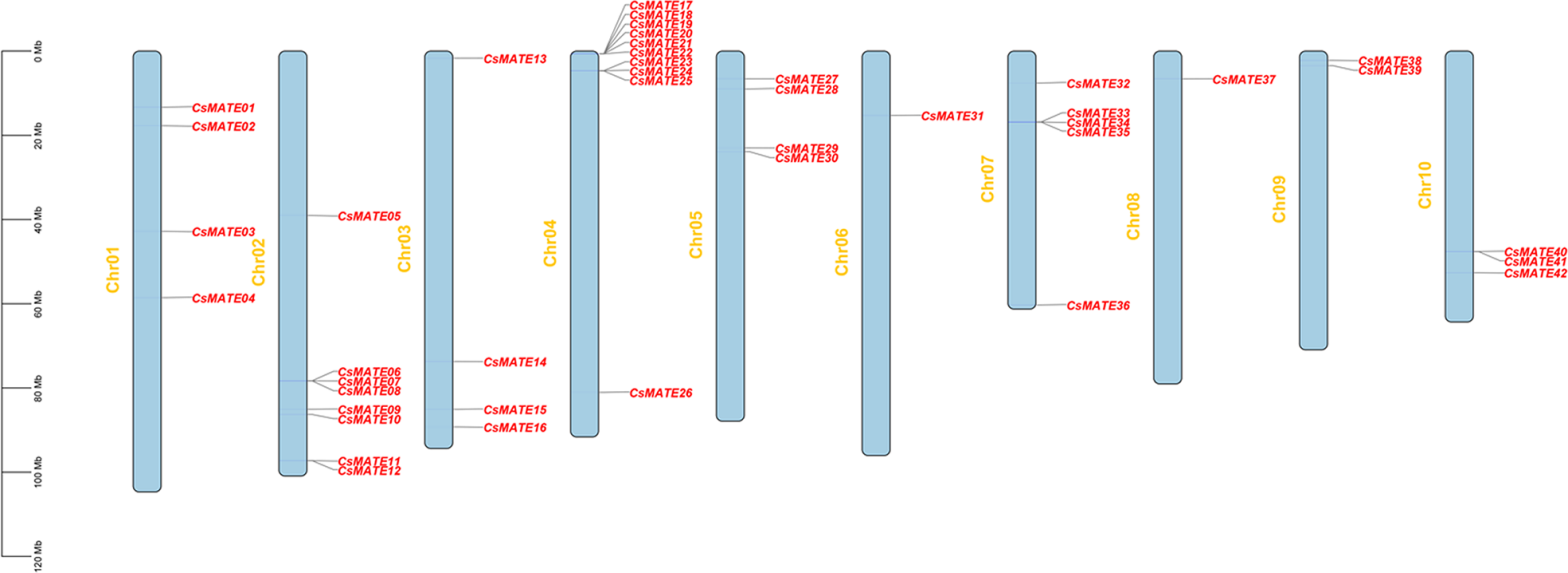
Distribution of *CsMATEs* on chromosomes of *C. sativa*. The y-axis indicates the length of chromosome in megabase (Mb).

We performed the homology search against the Arabidopsis MATE sequences and finally 42 CsMATE candidates were identified and named for their position on the chromosomes (**Table 1**). Forty-two *CsMATEs* are unevenly scattered on ten chromosomes, with chromosome four contains the highest number of *CsMATEs* and chromosome six contains the least (**Table 1** and **Figure 1**). It’s worth noting that twenty-one *CsMATEs* genes are physically adjacent to at least one another *CsMATEs*, such as *CsMATE6*–*CsMATE8*, *CsMATE11*–*CsMATE12*, *CsMATE17*–*CsMATE22*, *CsMATE2*3–*CsMATE25, CsMATE29*–*CsMATE30*, *CsMATE33*–*CsMATE35*, and *CsMATE40*–*CsMATE41* (**Figure 1**). Those *CsMATEs* account for ~50% of the total *CsMATE* genes. Moreover, near half of the *CsMATEs* are located in the proximal region of chromosome telomere (**Figure 1**).

**Table 1:** Detail information of CsMATEs.

Gene Name^a^	Gene ID^b^	Length^c^	pI^d^	MW^e^	SL^f^
*CsMATE01*	gene-LOC115702550	603	9.56	64111.87	Plas
*CsMATE02*	gene-LOC115705200	510	6.16	55448.57	Plas
*CsMATE03*	gene-LOC115711014	600	5.57	66053.91	Plas
*CsMATE04*	gene-LOC115712910	478	8.32	52682.78	Plas
*CsMATE05*	gene-LOC115704231	543	6.25	58847.88	Plas
*CsMATE06*	gene-LOC115705064	498	8.02	53766.41	Plas
*CsMATE07*	gene-LOC115704998	480	7.01	52855.52	Plas
*CsMATE08*	gene-LOC115704386	442	5.91	48420.02	Plas
*CsMATE09*	gene-LOC115703842	481	6.45	53039.56	Plas
*CsMATE10*	gene-LOC115704479	621	5.29	68007.98	Vacu
*CsMATE11*	gene-LOC115704728	501	6.59	54416.21	Vacu
*CsMATE12*	gene-LOC115707979	524	5.31	56805.39	Plas
*CsMATE13*	gene-LOC115708894	540	8.7	58085.01	Plas
*CsMATE14*	gene-LOC115708868	491	8.22	53432.55	Plas
*CsMATE15*	gene-LOC115710056	507	7.05	54875.91	Plas
*CsMATE16*	gene-LOC115708683	319	5.94	35375.83	Plas
*CsMATE17*	gene-LOC115714727	485	8.19	53346.36	Plas
*CsMATE18*	gene-LOC115714726	495	6.11	54439.4	Plas
*CsMATE19*	gene-LOC115714655	492	6.47	53938.45	Plas
*CsMATE20*	gene-LOC115712477	488	6.54	53419.89	Plas
*CsMATE21*	gene-LOC115714241	490	8.66	53946.72	Plas
*CsMATE22*	gene-LOC115714240	491	8.68	54434.61	Plas
*CsMATE23*	gene-LOC115714995	511	6.1	55863.48	Plas
*CsMATE24*	gene-LOC115712321	500	6.94	54840.62	Plas
*CsMATE25*	gene-LOC115714210	497	7.53	54268.94	Plas
*CsMATE26*	gene-LOC115713744	502	8.82	54664.57	Vacu
*CsMATE27*	gene-LOC115715674	497	5.12	54424.38	Vacu
*CsMATE28*	gene-LOC115715639	497	5.12	54388.58	Plas
*CsMATE29*	gene-LOC115715820	553	6	60358.2	Plas
*CsMATE30*	gene-LOC115717372	483	6.95	52314.4	Plas
*CsMATE31*	gene-LOC115719395	505	6.1	55712.17	Plas
*CsMATE32*	gene-LOC115723139	516	7.95	57018.9	Vacu
*CsMATE33*	gene-LOC115723617	484	8.94	52901.07	Plas
*CsMATE34*	gene-LOC115724138	473	6.81	51277.63	Plas
*CsMATE35*	gene-LOC115723312	500	6.41	54589.43	Plas
*CsMATE36*	gene-LOC115723477	490	5.76	54286.35	Plas
*CsMATE37*	gene-LOC115695936	502	7.01	53538.67	Vacu
*CsMATE38*	gene-LOC115697672	536	8.99	58082.75	Plas
*CsMATE39*	gene-LOC115698206	551	5.73	58931.1	Chlo
*CsMATE40*	gene-LOC115700595	513	5.97	55800.43	Plas
*CsMATE41*	gene-LOC115699384	506	6.98	54837.94	Plas
*CsMATE42*	gene-LOC115701322	526	6.53	57241.14	Plas

a Gene named for their position on the chromosomes.

b Accession number of *C. sativa* locus ID.

c Protein length in amino acid.

d Isoelectric points.

e Molecular weight in Dalton.

f Subcellular localization, plas: plasm membrane, Chol: chloroplast, Vacu: vacuole.

We also predicted the physical-chemical properties and subcellular localization of the CsMATEs and found that the longest MATE is CsMATE10, consisting of 621 amino acids, while the shortest CsMATE16 is comprised of 319 amino acids (**Table 1**). The isoelectric points (pI) and molecular weights (MW) of CsMATEs range from 5.12–9.56 and 35.38–68 kilodalton (kDa), respectively (**Table 1**). Thirty-five CsMATEs were localized in the plasma membrane, six in the vacuole, and only CsMATE39 localized in the chloroplast (**Table 1**).

**Figure 2:**
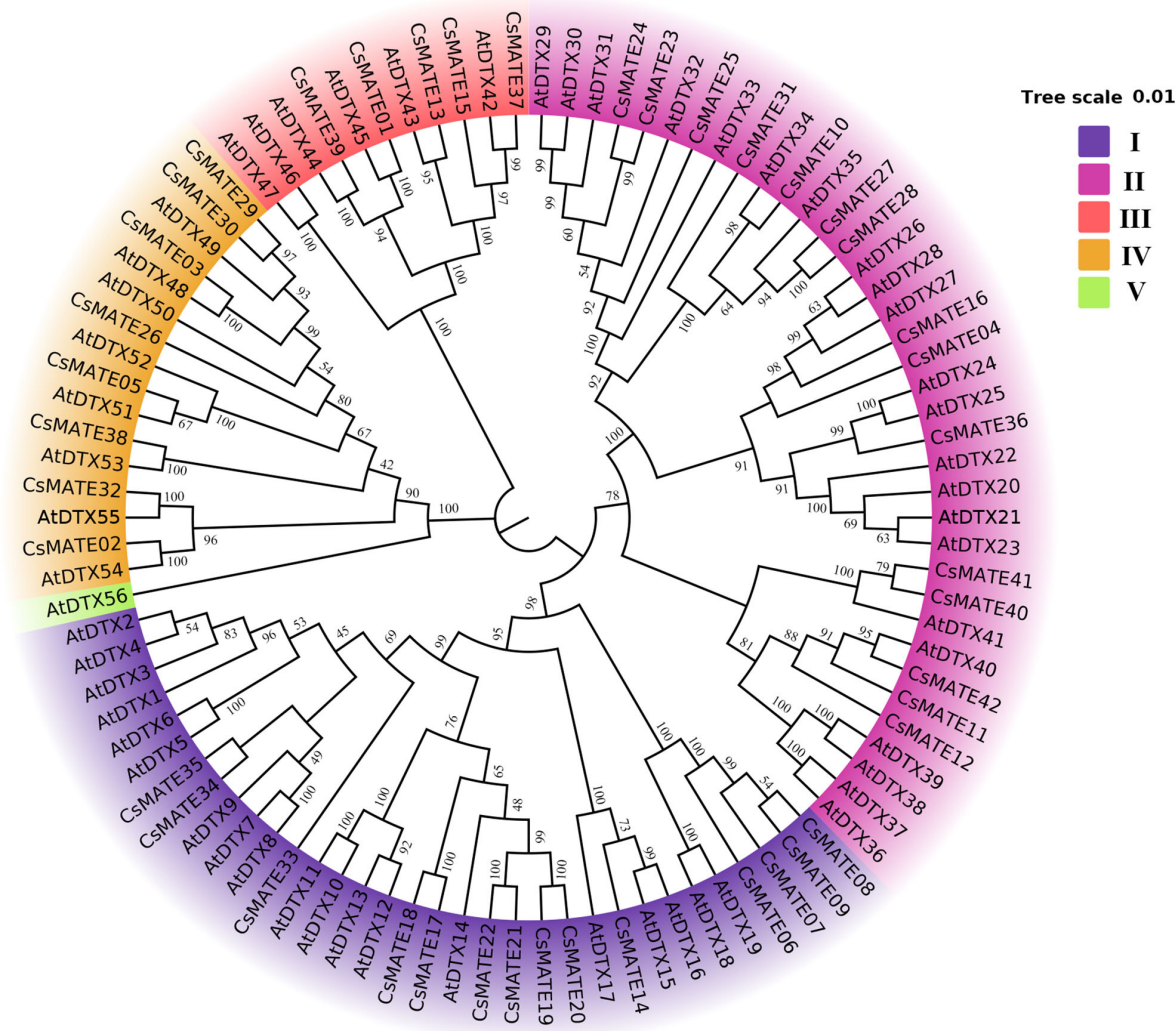
Phylogenic tree of MATE proteins in *Cannabis sativa* (CsMATEs) and *Arabidopsis thaliana* (AtDTXs). Phylogenic tree was built using MEGA 7.0 software neighbor-joining method (Kumar et al., 2016) with a bootstrap analysis of 1000 replicates.

To investigate of the evolutionary relationship of the CsMATEs, we constructed an interspecifc phylogenic tree using *C. sativa* and *A. thaliana* MATEs sequences with phylogenetic inference of neighbor-joining (**Figure 2**). The topology of the phylogenic tree divides CsMATEs into four major subfamilies. Subfamily I contains 14 CsMATEs, subfamily II has 15 CsMATEs, subfamily III possesses five CsMATES, and subfamily IV owns eight CsMATEs (**Figure 2**). Importantly, physically adjacent *CsMATEs* were all clustered in the same subfamily (**Figure 1** and **Figure 2**), which indicates tandem duplication is the major evolutionary driving force for *CsMATEs* expansion (Wang et al., 2019).

### Conserved motifs of CsMATEs and gene structure of *CsMATEs*

**Figure 3:**
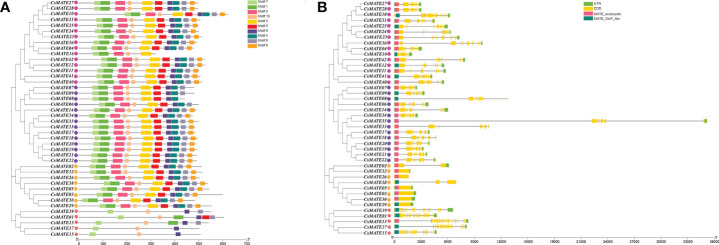
Conserved motifs and gene structure of CsMATEs. **(A)** CsMATEs Phylogenic tree and motifs distributions. Motifs are indicated as top right. **(B)** Gene structure and domain distribution. UTR, untranslated region; CDS, coding region; yellow boxes, exons; gray lines, introns; pink boxes, MATE eukaryotic domains; dark green boxes, DinF like domain. Purple dots indicates CsMATE from subfamily I; rose red dots, Subfamily II; pink dots, subfamily III; yellow dots, subfamily IV.

Conserved protein motifs are associated with gene function and protein subcellular localizations. We isolated 10 predicted conserved motifs (**Supplementary Figure S1**) using MEME (Bailey et al., 2006) and studied their distributions within CsMATEs. Phylogenetic analysis grouped CsAMTEs into four subfamilies, which is consistent with the interspecific phylogenetic tree in **Figure 2** (**Figure 3A**). We found that 32 CsMATEs (76% of total identified) from subfamily I, II and IV included all 10 motifs and those motifs shared the same order. CsMATE6, 7 and 9 were lack of motif 8; CsMATE8 is without of moitf6 and motif8. CsMATEs from subfamily III had motifs less than six, with CsMATE15 and CsMATE17 only containing motifs 7, 9 and 10 (**Figure 3A**).

To further examine the evolutionary lineages of *CsMATEs*, we compared the gene structure of *CsMATEs*. The results showed that phylogenetically close *CsAMTEs* shared the same exon number, length and composition (**Figure 3B**). For instance, *CsMATEs* from subfamily IV had no intron except for *CsMATE2* and *CsMATE38* that contained one and two introns, respectively. *CsMATEs* from subfamily III owned most exons of at least 10. A longest intron that over 20 kb was found in *CsMATE35* from subfamily I (**Figure 3B**). In addition, as tandem duplication is the main force of *CsMATEs* expansion, physically adjacent *CsMATEs* also showed same exon/intron pattern, such as *CsMATE6*–*CsMATE8* and *CsMATE2*3–*CsMATE25* (**Figure 1** and **Figure 3B**). Although gene structures between different subfamilies were divergent, we found two crucial domains that closed to 5’ end of the gene bodies (**Figure 3B**).

### Expression pattern of *CsMATEs* in deferent tissues

**Figure 4:**
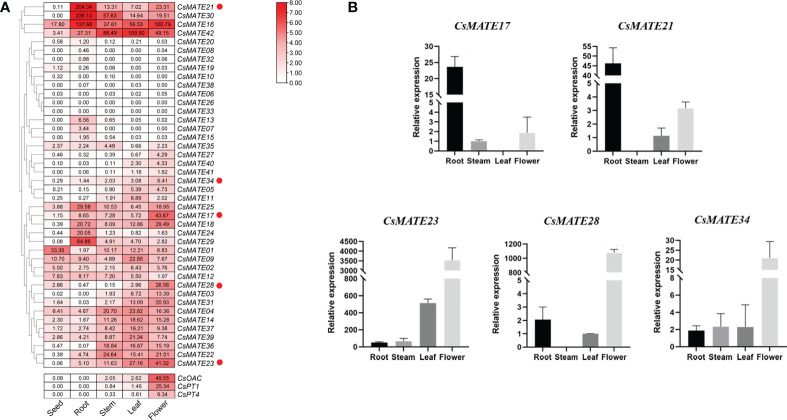
Expression of *CsAMTEs* and cannabinoid biosynthetic genes. **(A)** Expression profile of *CsMATEs* in seed, root, stem, leaf and flower. PFKM values were used to configure the heatmap. Red dots, genes selected for expression verification. *CsOAC*, *olivetolic acid synthase*; *CsPT1* and *CsPT4*, *prenyltransferase 1* and *4* gene, repectively. **(B)** quantitative reverse transcription PCR verification. y-axis, relative gene expression normalized to *CsEF1-alpha*. x-axis, different tissues. Data, mean ± SD.

Gene expression pattern is to some extent indicative for its potential function, especially for transporter proteins that mostly interact with substrates in tissues where metabolites are synthesized (Nogia and Pati, 2021). We visualized the *CsMATEs* expressions from different tissues of hemp variety Dinamed Kush, a species with high cannabinoids content, using FPKM (Fragments Per Kilobase of exon model per Million mapped fragments) and cluster analysis, and indeed found the divergent expression patterns (**Figure 4A**). Specifically, only *CsMATE1* showed higher expression in seed compared with other tissues, *CsMATE7*, *CsMATE13*, *CsMATE21*, *CsMATE24*, *CsMATE25*, *CsMATE29* and *CsMATE30* exhibit relatively strong expression in root, *CsMATE22* and *CsMATE36* have the moderate stronger expression in stem, transcripts of *CsMATE4*, *CsMATE9*, *CsMATE11*, *CsMATE14*, *CsMATE37*, *CsMATE39* and *CsMATE42* are abundantly detected in leaf, and *CsMATE03*, *CsMATE05 CsMATE17*, *CsMATE23*, *CsMATE28*, *CsMATE31*, *CsMATE34* and *CsMATE4*0 are the genes that expressed mainly in flowers (**Figure 4A**).

As we are interested in CsMATEs that involved in transportation of cannabinoids, and to further verify the accuracy of the transcriptomic data, we analyzed the expressions of representative MATE genes by qRT-PCR (real-time quantitative reverse transcription PCR) including *CsMATE17*, *CsMATE23*, *CsMATE28* and *CsMATE34* which had a highest expression in flower but also expressed moderately in stem and leaf according to transcriptome data, as well as *CsMATE21* that showed strong root specificity in transcriptomic heatmap (**Figure 4A**). *CsMATE17*, unexpectedly, was predominantly expressed in root, inconsistent with the RNA-seq data, while the other four gene showed the same expression pattern as in RNA-seq (**Figure 4B**). Additionally, we also examined the expression patterns of cannabinoid biosynthetic pathway genes including *CsOAC*, *CsPT1 and CsPT4* (**Figure 4A**). The result showed that *CsMATE23*, *CsMATE28* and *CsMATE34* exhibited similar expression patterns with cannabinoids synthetic genes, suggesting those genes might involve in cannabinoids synthesis (**Figure 4A**).

### Expression of alternative splicing isoforms of C*sMATEs* and *cis*-elements in *CsMATE23*, *CsMATE28* and *CsMATE34*

**Figure 5:**
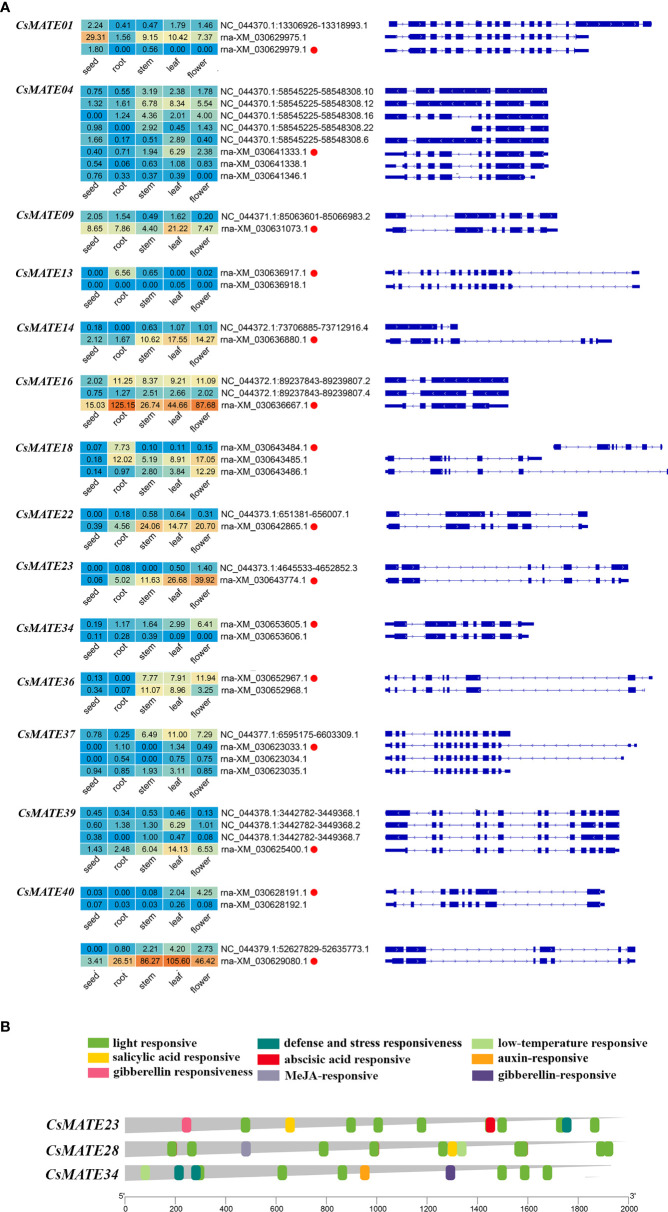
Expression of alternative splicing isoforms **(A)** and *cis*-acting elements in *CsMATEs* **(B)**. Red dots, alternative splicing isoform used for analysis in this study. Heatmap was configured using PFKM value. Length of gene promoters in bp.

Alternative splicing is widespread in plants as a transcriptional regulatory mechanism that allows a single gene to encode a variety of different transcripts and protein products (Roy et al., 2013). To further understand the transcriptional mechanisms of *CsMATEs*, we performed alternative splicing analysis. We observed 15 *CsMATEs* have alternative splicing events, accounting for about 1/3 of *CsMATEs*, with a total of 43 alternative splicing isoforms. Numbers of alternative splicing isoforms of a single *CsMATE* range from one to seven, and most of the alternative splicing isoforms form the same pre-mRNA were differentially expressed in the same tissues. Of note, although *CsMATE23* and *CsMATE34* both have an alternative splicing isoform, the alternative splicing isoforms barely express in the tissues we examined (**Figure 5A**).

Light is known to affect the content of cannabinoids (Eichhorn et al., 2019). The *cis*-acting elements in the promoter region (2 kb upstream of ATG codon) of *CsMATE23*, *CsMATE28* and *CsMATE34* were analyzed using plantCARE software (Lescot et al., 2002). We found that the most abundant *cis*-acting element is the light responsive element, and at least six light responsive *cis*-acting elements are found in each of the *CsMATE23*, *CsMATE28* and *CsMATE34* promoter regions (**Figure 5B**). Phytohormones responsive *cis*-acting elements are the second abundant found in their promoter regions (**Figure 5B**). We also revealed one and two defense and stress responsive *cis*-acting elements in *CsMATE17* and *CsMATE28* promoters, respectively, and one low temperature-responsive *cis*-element in *CsMATE23* promoter (**Figure 5B**).

### Cannabinoids contents, transcription of *CsMATE23*, *CsMATE28* and *CsMATE34* were affected under UV-B light

As we found numerous light responsive c*is*-acting elements in the promoters of *CsMATE23*, *CsMATE28* and *CsMATE34* (**Figure 5B**) and lights are known to affect accumulation of cannabinoids (Eichhorn et al., 2019), we then studied the content of cannabinoids, cannabinoids biosynthetic intermediates and the expressions of *CsMATE23*, *CsMATE28* and *CsMATE34* under UV-B light treatment.

**Figure 6:**
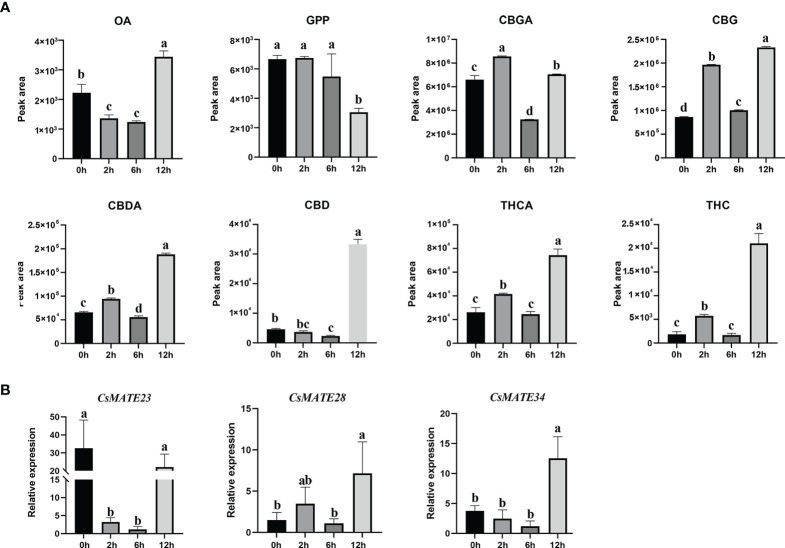
Cannabinoids contents **(A)** and expression of *CsMATE23*, *CsMATE38* and *CsMATE34* under UV-B light **(B)**. Leaves from UV-B treated three-weeks-old *C. sativa* seedlings were collected at 0, 2, 6 and 12 hours. Cannabinoids contents were measured using QQQ MS/MS. gene expressions were detected by qRT-PCR with *CsEF1-alpha* as internal reference. Three biological repeats were performed. Different letters above the bars indicate significantly different values (*p* < 0.05) calculated using one-way analysis of variance (ANOVA) followed by Tukey’s multiple range test.

Three-weeks-old cannabis seedlings were subjected to UV-B light treatment, and quantitative analysis of cannabinoid contents from leaves was performed by QQQ-MS/MS. In summary, content of OA decreased significantly after two hours or six hours UV-B treatment and reached highest at 12 hours, whereas we only observed a decrease of GPP at 12 hours (**Figure 6A**). Except for CBD that showed a decrease at two hours treatment and an increase after 12 hours UV-B treatment, the contents of CBGA, CBG, THCA, THC and CBDA increased after two hours, then decreased at six hours, reached the highest at 12 hours (**Figure 6A**).

Correspondingly, expressions of *CsMATE23*, *CsMATE28* and *CsMATE34* also exhibited significant changes under UV-B treatment (**Figure 6B**). *CsMATE23* was greatly reduced after two hours UV-B treatment and restored its expression at 12 hours. The expression of *CsMATE28 and CsMATE34* displayed no significant change during the first six hours treatment, but significantly increased at 12 hours. (**Figure 6B**).

## Discussion

MATEs have been identified in a variety of plant species, including 56 MATEs in Arabidopsis, 55 in rice (Wang et al., 2016). 67 in tomato (Santos et al., 2017), 64 in potato (Huang et al., 2021), 70 in *Medicago truncatula* (Wang et al., 2017), 72 in cotton (Xu et al., 2019) and 117 in soybean (Liu et al., 2016). In the present study, we identified 42 CsMATEs and investigated their physical-chemical properties, gene distribution, evolutionary relationships, conserved motifs, gene structures and gene expressions. CsMATEs contains only 42 members (**Table 1**) and is the fewest when compared with other plant species, which suggests CsMATEs may have undergone contraction during evolution. Those 42 CsMATEs were divided into four subfamilies and distributed on 10 chromosomes, with ~50% *CsMATEs* adjacent to at least one another *CsMATEs* (**Figure 1**), thus the expansion of *CsMATEs* might be largely due to tandem duplication (Wang et al., 2019).

Although cannabinoids synthetic pathways have already been illustrated, the translocation of the cannabinoids and biosynthetic intermediates are not known (Güllck and Moller, 2020). This inevitably hinders cannabinoids heterologous biosynthesis (Güllck et al., 2020). Cannabinoids are synthesized in glandular trichomes and flowers of female *C. sative* and their biosynthetic processes undergo cellular compartmentalization (Güllck and Moller, 2020). For instance, plastid localized CsaPT4 prenylates cytosolic synthesized OA in chloroplast to yield CBGA (Taura et al., 2009; Gagne et al., 2012; Luo et al., 2019; Güllck et al., 2020). Metabolite transporter proteins that could shuttle from cytosol to chloroplast should exist in *C sativa* cells. However, subcellular localization prediction indicates that vast majority of CsMATEs locate in vacuole or plasma membrane (**Table 1**). Although CsMATE39 contains putative chloroplast localization signal (**Table 1**), it expresses mainly in leaf (**Figure 4A**), the tissue that is not the most abundant cannabinoids accumulate. We assume that transporters from other families might be involved in translocating phenolic OA such as ATP binding cassette transporters which could translocate plant phenolic metabolites (Hwang et al., 2016). THCAS is secreted to apoplastic space of the glandular trichome to carry out oxidative cyclization of CBGA (Sirikantaramas et al., 2005). The plastid produced CBGA thus should be translocated across the plasm membrane. Thirty-five CsMATEs are predicted to localized on plasm membrane (**Table 1**). Of them, *CsMATE03*, *CsMATE05*, *CsMATE23*, *CsMATE28*, *CsMATE31*, *CsMATE34* and *CsMATE40* had a highest expression in flower but also expressed moderately in stem and leaf (the tissues that have glandular trichomes) according to transcriptome data (**Figure 4A**) and qRT-PCR (**Figure 4B**). Although we don’t have the trichome-specific transcriptional data of those seven genes in variety DK, they uniformly expressed in the trichomes of the other nine *C. sativa* varieties (Zager et al., 2019, **Supplementary Figure S2**). Moreover, the seven genes exhibited similar expression pattern in different tissues as that of cannabinoid synthetic genes (**Figure 4A**), which further indicates the involvement CsMATEs in biosynthesis of cannabinoids.

Within the seven genes, except for *CsMATE23*, *CsMATE28*, *CsMATE34* and *CsMATE40* all have an unexpressed alternative spliceform in tissues we studied (**Figure 5A**). Light responsive *cis*-acting elements were abundantly detected in promoters of the representative *CsMATEs* (*CsMATE23*, *CsMATE28* and *CsMATE34*), reflects those genes could be regulated by lights. UV-B is known to affect accumulation of cannabinoids (Lydon et al., 1987). In accordance with this, the contents of cannabinoids and corresponding intermediates were changed under UV-B treatment (**Figure 6A**). As expected, the variation trend of CBGA was analogous to its direct downstream THCA, CBDA and CBG (**Figure 6A**), reflects the precursor nature of the CBGA (Güllck et al., 2020). Notably, although overall variation of OA is similar to CBGA, accumulation of OA is opposed to CBGA at two hours treatment where OA content was decreased but CBGA contents was increased compared to zero hours treatment (**Figure 6A**). Meanwhile, GPP did not show any change at two hours treatment (**Figure 6A**). Therefore, increase of CBGA after two hours UV-B treatment could be largely caused by over-usage of OA for synthesizing CBGA either due to enhanced CBGAS activity or more efficient translocation of OA form cytosol to plastid. Unfortunately, none of the *CsMATEs* we tested was up-regulated after two hours treatment (**Figure 6B**). We reasoned that plastid located ATP binding cassette transporters that translocate phenolic metabolites might responsible for OA transportation (Hwang et al., 2016). Moreover, Although the transcriptional variation of *CsMATE23* is analogous to the content of OA (**Figure 6B**), OA is synthesized in cytosol and CsMATE23 is localized in plasma membrane (**Table 1** **)**. Meanwhile, *CsMATE23* is barely transcribed in *C sativa* root, however its Arabidopsis ortholog AtRHS2 (AtDTX31) located in plasma membrane of root is required for hairy root elongation (Won et al., 2009). Hence, we may also exclude participation of CsMATE23 in transporting OA precursors.

The most interesting finding we observed is the correlation between transcriptional alterations of *CsMATEs* with the accumulation of CBGA, THCA and CBDA (**Figure 6**). As the precursor of THCA and CBDA, CBGA content did not show much alteration at 12 hours treatment compared to the control zero hour (**Figure 6A**). However, the contents of THCA, THC, CBDA and CBD were all increased at least more than four times at 12 hours compared to zero hours and transcriptions of *CsMATE28* and *CsMATE34* were also significantly enhanced at 12 hours (**Figure 6B**). This could be explained by that increased CsMATE28 or CsMATE34 more inefficiently transport CBGA to the apoplastic space to enable its subsequent conversions (Sirikantaramas et al., 2005).

## Conclusion

We identified 42 CsMATEs and analyzed their structural features, evolutionary relationships and expression patterns. We found number of CsMATEs was subjected to contraction and its expansion within the family was mainly due to tandem duplication. Though RNA-seq and qRT-PCR analysis, we found two root-specifically transcribed *CsMATEs* (*CsMATE17* and *CsMATE27*) and three *CsMATEs* (*CsMATE23*, *CsMATE28* and *CsMATE34*) whose transcription pattern were correlated with transcriptions of cannabinoids biosynthetic genes. In addition, although CBGA content was not much affected under UV-B treatment at 12 hours, accumulations of THCA, CBDA and CBG were increased. This could be due to the increased expression of *CsMATE28* or *CsMATE34*. Although multiple evidences suggest that CsMATEs may play an important role in the synthesis and transport of cannabinoids, the transport of cannabinoids is a complex process. Therefore. The functions of candidate genes (CsMATE28 and CsMATE34) in cannabinoid transportations should be investigated in depth in the future.

## Data availability statement

The original contributions presented in the study are included in the article/**Supplementary Material**. Further inquiries can be directed to the corresponding authors.

## Author contributions

WC and HW designed the study. WC, HW, SW, XC and XM wrote the manuscript. SW, XC, XM, YW, AW, MA and QD conducted the bioinformatic analysis. XC and XM performed the qRT-PCR and UV-B treatment. All authors contributed to the article and approved the submitted version.

## Funding

This work is supported by Scientific and technological innovation project of China Academy of Chinese Medical Sciences (CI2021A04113).

## Conflict of interest

The authors declare that the research was conducted in the absence of any commercial or financial relationships that could be construed as a potential conflict of interest.

## Publisher’s note

All claims expressed in this article are solely those of the authors and do not necessarily represent those of their affiliated organizations, or those of the publisher, the editors and the reviewers. Any product that may be evaluated in this article, or claim that may be made by its manufacturer, is not guaranteed or endorsed by the publisher.
