# Supplementary material for: Characterization and expression analysis of MATEs in Cannabis sativa L. reveals genes involving in cannabinoid synthesis
Source: Front Plant Sci. 2022 Oct 13;13:1021088. doi: 10.3389/fpls.2022.1021088 (PMC9606718; doi:10.3389/fpls.2022.1021088)
Supplement: Supplementary file 1 [file DataSheet_1.docx]

| **Supplementary Table 1. Primers for qRT-PCR** | |  |
| --- | --- | --- |
| Gene | Forward primer sequences (5’-3’) | Reverse primer sequences (5’-3’) |
| *CsMATE17* | GCCATATCTCTTTCTGGGGTC | AAGCCCAAGTTTCTTGTATTGCT |
| *CsMATE21* | CTCTTTCTAGTACAGCTATTGCGA | CCCGTGTTGGTTAGGATTCCG |
| *CsMATE23* | TATGCCAATACTCCCTCGGC | AGTTTCCAAAGCACTC |
| *CsMATE28* | TGGCATCAATTCCCTCACCA | TCCAACGCACTTCCCATTCC |
| *CsMATE34* | CTCTTGCAGGTTGTCTCCGT | TAAAGTCTCCAATCCACCCGC |
| EF1-α | ACCAAGATTGACAGGCGTTC | CCTTCTTCTCCACAGCCTTG |

**Supplementary figure legends**

**Supplementary Figure 1**. Sequences of conserved motifs.

**Supplementary Figure 2**. *CsMATEs* expression profile in trichomes of nine *C. sativa* varieties. Transcriptional data were downloaded from NCBI website (Zager et al, 2019).
