# Supplementary figures and images for: Characterization and expression analysis of MATEs in Cannabis sativa L. reveals genes involving in cannabinoid synthesis
Source: Front Plant Sci. 2022 Oct 13;13:1021088. doi: 10.3389/fpls.2022.1021088 (PMC9606718; doi:10.3389/fpls.2022.1021088)

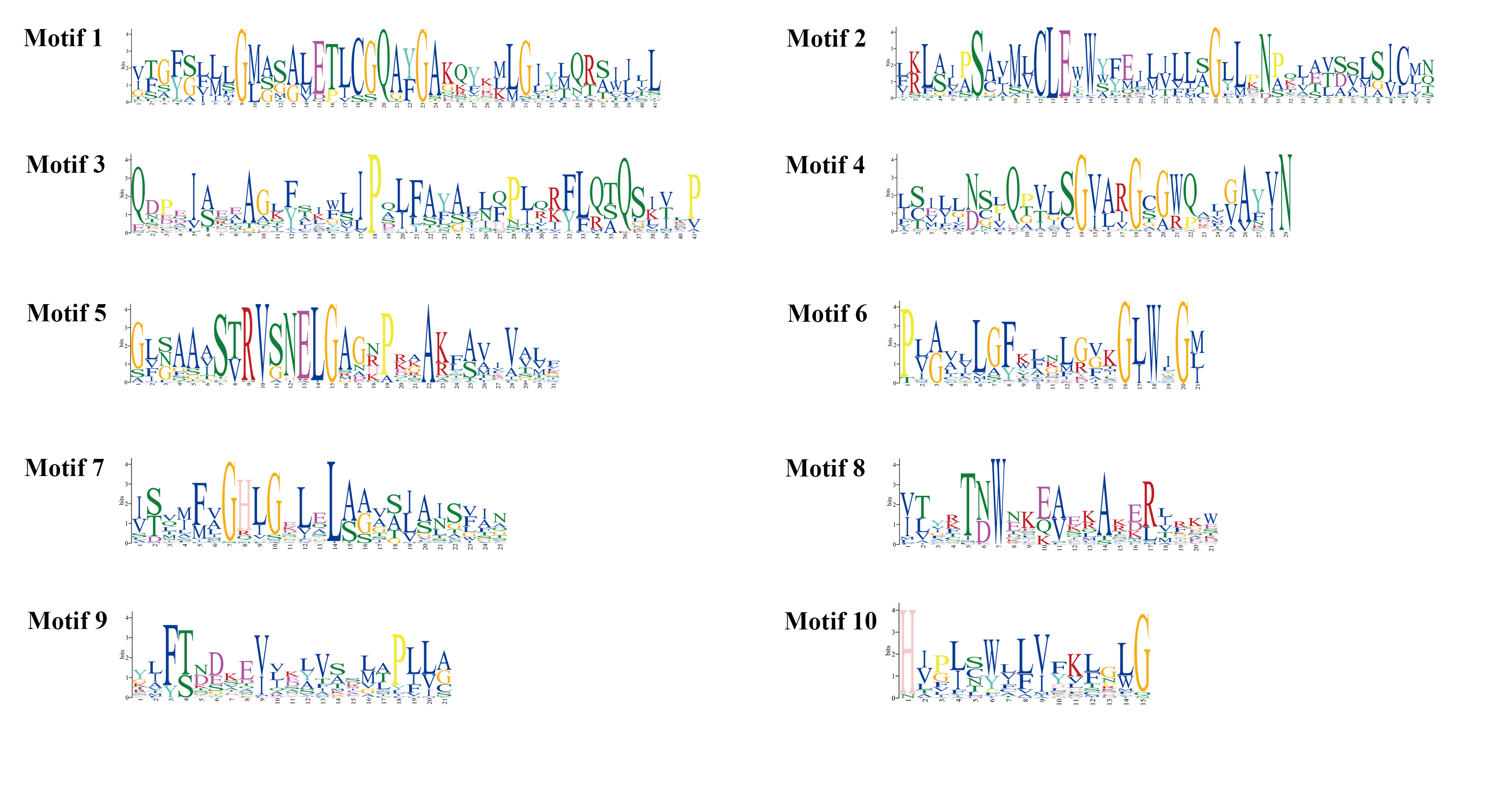

Supplement: Supplementary file 2 [file Image_1.tif]

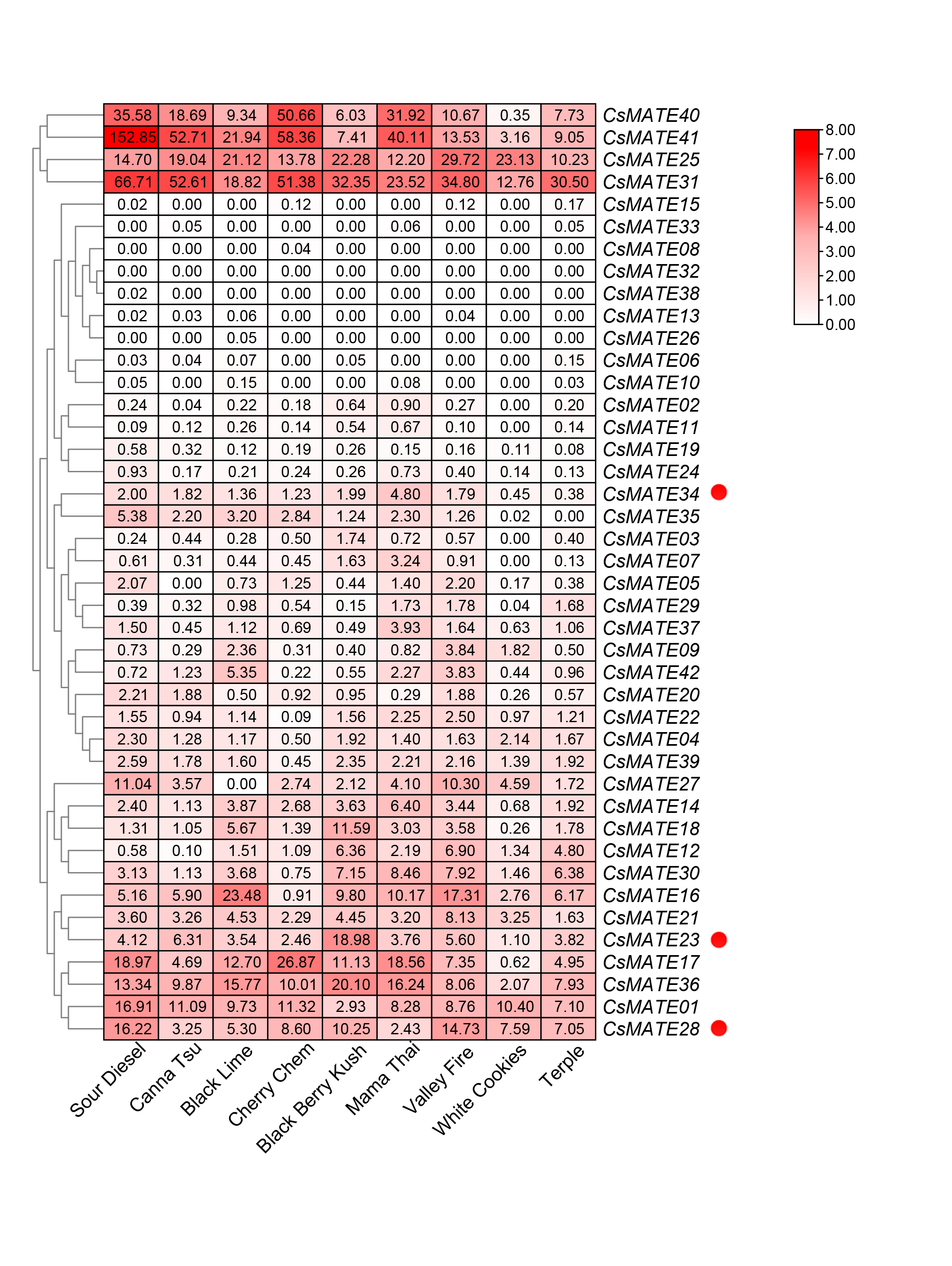

Supplement: Supplementary file 3 [file Image_2.tif]
